# Supplementary material for: Accuracy of haplotype estimation and whole genome imputation affects complex trait analyses in complex biobanks
Source: Commun Biol. 2023 Jan 26;6:101. doi: 10.1038/s42003-023-04477-y (PMC9876938; doi:10.1038/s42003-023-04477-y)
Supplement: Supplementary file 2 — Supplementary Information [file 42003_2023_4477_MOESM2_ESM.pdf]

Accuracy of haplotype estimation and whole genome imputation affects complex trait analyses in complex biobanks

Supplementary Information

Vivek Appadurai[1,2 *], Jonas Bybjerg-Grauholm[2,3], Morten Dybdahl Krebs[1,2], Anders Rosengren[1,2], Alfonso Buil[1,2], Andrés Ingason[1,2], Ole Mors[2,4], Anders D. Børglum[2,5,6], David M. Hougaard[2,3], Merete Nordentoft[2,7,8], Preben B. Mortensen[2,9,10], Olivier Delaneau[11], Thomas Werge[1,2], Andrew J. Schork[1,2,12 *]

1. Institute of Biological Psychiatry, Mental Health Center Sankt Hans, Roskilde, Denmark 4000
2. The Lundbeck Foundation Initiative for Integrative Psychiatric Research, iPSYCH, Aarhus, Denmark
3. Danish Center for Neonatal Screening, Statens Serum Institut, Copenhagen, Denmark
4. Psychosis Research Unit, Aarhus University Hospital - Psychiatry, Aarhus, Denmark
5. Department of Biomedicine and Center for Integrative Sequencing, iSEQ, Aarhus University, Aarhus, Denmark
6. Center for Genomics and Personalized Medicine, CGPM, Aarhus University, Aarhus, Denmark
7. Mental Health Services in the Capital Region of Denmark, Copenhagen, Denmark
8. Department of Clinical Medicine, Faculty of Health Sciences, University of Copenhagen, Copenhagen, Denmark
9. NCRR - National Center for Register-Based Research, Business and Social Sciences, Aarhus University, Aarhus, Denmark
10. CIRRAU - Centre for Integrated Register-Based Research, Aarhus University, Aarhus, Denmark
11. Department of Computational Biology, University of Lausanne, Lausanne, Switzerland
12. The Translational Genomics Research Institute, Phoenix AZ, USA

* Corresponding Authors: (vivek.appadurai@regionh.dk, andrew.joseph.schork@regionh.dk)

Parental Birthplace	iPSYCH2012	iPSYCH2015i
Denmark	67044	41673
Denmark_Europe	2416	1591
Denmark_Scandinavia	1476	913
Europe	1169	785
Denmark_Unknown	829	543
MiddleEast	775	563
Asia	594	384
Asia_Denmark	581	292
Africa	473	277
Denmark_Greenland	435	284
Africa_Denmark	431	292
Denmark_NorthAmerica	363	234
Denmark_MiddleEast	354	216
Denmark_SouthAmerica	235	184
Scandinavia	109	67

Supplementary Table 1. Ancestry composition of iPSYCH by parental birthplace as obtained from the Danish Civil Registers[1]. Underscore delimited combinations indicate parents born in different regions.

Supplementary Figure 1. Variation in phasing accuracy and SNP density across 22 chromosomes

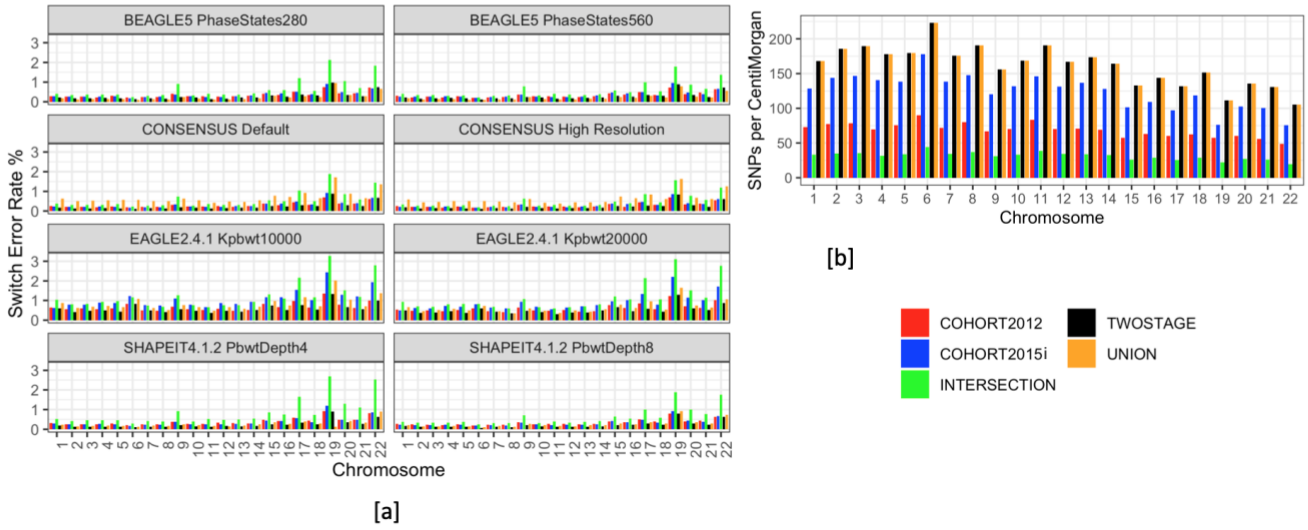

[a] Haplotype estimation accuracy as shown by switch error rates obtained from comparing computationally assigned phase to mendelian transmission in 124 trio offspring whose parental genotypes are known. [b] SNP density across chromosomes within each data integration protocol. (Cohort2012 in separate protocol: red bars, Cohort 2015i in separate protocol: blue bars, intersection: green bars, twostage: black bars, union: orange bars)

Supplementary Note 1:

Imputation accuracy within personal genomes project – UK samples

BAM files corresponding to 10 samples from the personal genomes project - UK[2] were downloaded from the European Genome-Phenome Archive (EGA, study accession: PRJEB17529, sample accessions: SAMEA4545245, SAMEA4545246, SAMEA4545247, SAMEA4545248, SAMEA4545249, SAMEA4545250, SAMEA4545251, SAMEA4545252, SAMEA4545253, SAMEA4545254). Variant calling was performed using samtools mpileup and the samples were further down sampled to each of the two iPSYCH genotyping arrays and added to cohorts arising from each data integration protocol prior to phasing and imputation. The accuracy of the imputation was calculated as the squared Pearson correlation coefficient between the imputed dosages and variant calls at 6.5 million loci not genotyped on either iPSYCH array. The results as shown in Supplementary Figures 2a, b across minor allele frequency bins as ascertained from the HRCv1.1 haplotype reference panel show similar results to the results obtained by gauging the accuracy at the 10,000 SNPs masked prior to phasing. The accuracy of imputation appears to rely more on choice of data integration protocol than haplotype estimation tool. The haplotypes obtained from SHAPEIT4.1.2 in presence of high missingness introduced by the union protocol led to inaccurate imputations.

A comparison of imputation accuracy between the two iPSYCH genotyping arrays as shown in Supplementary Figure 2c reveals that all tools yield more accurate imputations in the cohort generated using the denser Illumina global screening array v2.0, despite a relatively lesser sample size for haplotype estimation as compared to the cohort generated using the Infinium Psych Chip v1.0 with less dense SNP information but a higher sample size.

Supplementary Figure 2. Imputation accuracy in personal genomes project - UK samples.

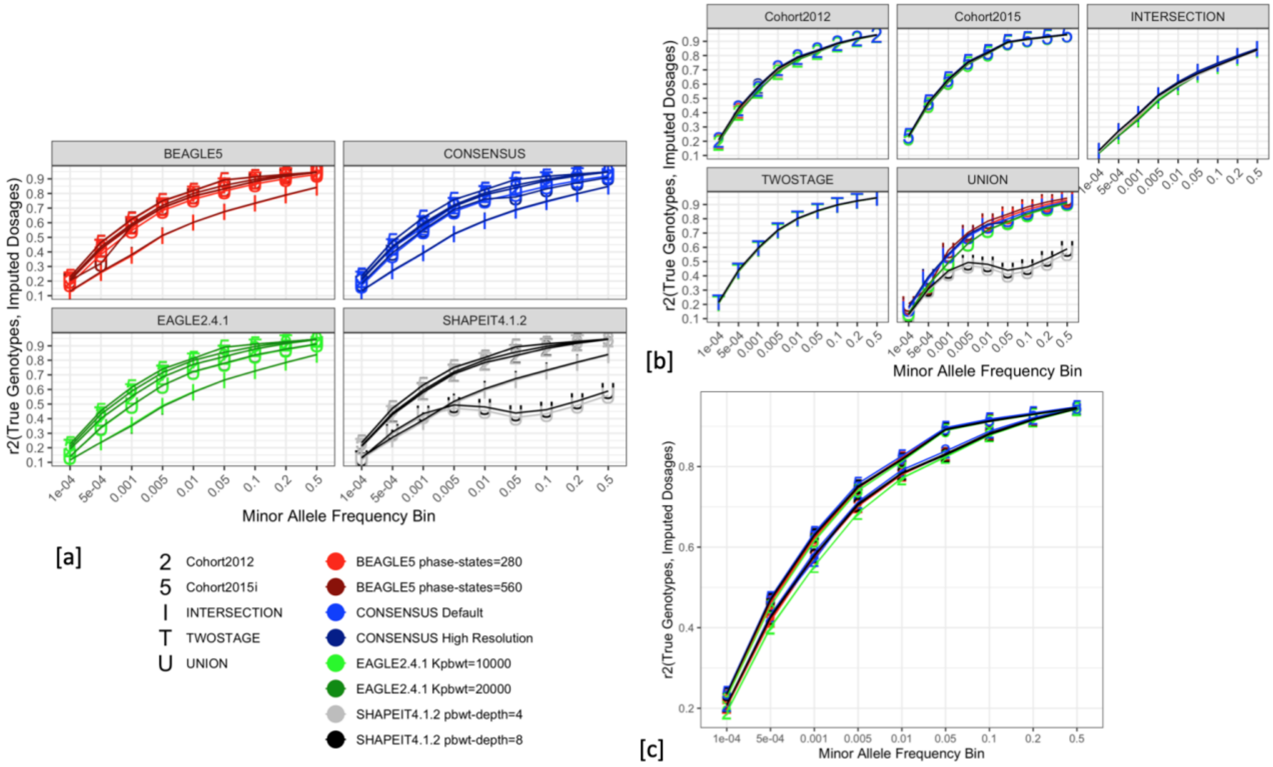

Accuracy of imputation within the 10 personal genomes project - UK whole genome sequenced samples, calculated as the squared Pearson correlation coefficient between imputed dosages and true genotypes at loci not present on either iPSYCH genotyping array. [a] Grouped by choice of haplotype estimation tool. [b] Grouped by choice of data integration protocol. [c] Comparison between imputation accuracy obtained by using each iPSYCH genotyping array. (Cohort 2012 denoted by shape "2", Cohort 2015i denoted by shape "5", intersection protocol denoted by shape "I", twostage protocol denoted by shape "T" and union protocol denoted by shape "U". Light Red: Beagle5 Phase-States=280, Dark Red: Beagle5 Phase-States=560, Light Green: Eagle2.4.1 at Kpbwt=10000, Dark Green: Eagle2.4.1 at Kpbwt=20000, Gray: Shapeit4.1.2 pbwt-depth=4, Black: Shapeit4.1.2 pbwt-depth=8, Light Blue: Consensus across all three tools at default parameters, Dark Blue: Consensus across all three tools at higher resolution parameters).

Supplementary Note 2

As the pre-phasing stage prior to imputation deals with deficiencies in the data such as systemic missingness and sparsity of genotype information, and relying on the prevailing wisdom from tool developers that imputation methods have reached a saturation for accuracy[5] and therefore compete by improving user friendliness and enhancing computational efficiency, most of the analysis in this paper chose to empirically estimate the variation between phasing methods across data integration protocols and consistently use BEAGLE5.1 for imputations. However, in Supplementary Figure 5 and Supplementary Table 11, we present a comparison of imputation accuracies between Impute5[5], Minimac4[6] and BEAGLE5.1[7] at 117 masked loci on chromosome 22, across 129,851 individuals, totaling 15,035,934 observations. In each case, we use haplotypes pre-phased by BEAGLE5.1 and the haplotype reference consortium HRCv1.1 dataset as the reference. All tools were run using default parameter settings and while all three methods deliver similar performance at common allele frequencies in well performing data integration scenarios (r^2 ~ 0.9 - 0.94 for separate, twostage protocols), at rarer allele frequencies (MAF < 0.01) and in less optimal data integration scenarios, BEAGLE5.1 and Minimac4 tended to outperform Impute5, with the most notable difference occurring in the *intersection* protocol. With a sparse backbone to impute from, BEAGLE5.1 delivers an r^2 improvement of ~ 0.05 in comparison to other tools, even at common allele frequencies. The tools varied by degrees of user friendliness, as BEAGLE5.1 necessitated imputing the iPSYCH cohort in batches of 10,000 samples each, while Impute5 requires the target region to be divided into chunks with a buffer, imputed and then concatenated together using bcftools. Minimac4 performed the chunking internally but required converting the reference haplotypes from VCF to m3VCF format using Minimac3.

Supplementary Figure 3. Imputation accuracy compared across different tools and data integration protocols.

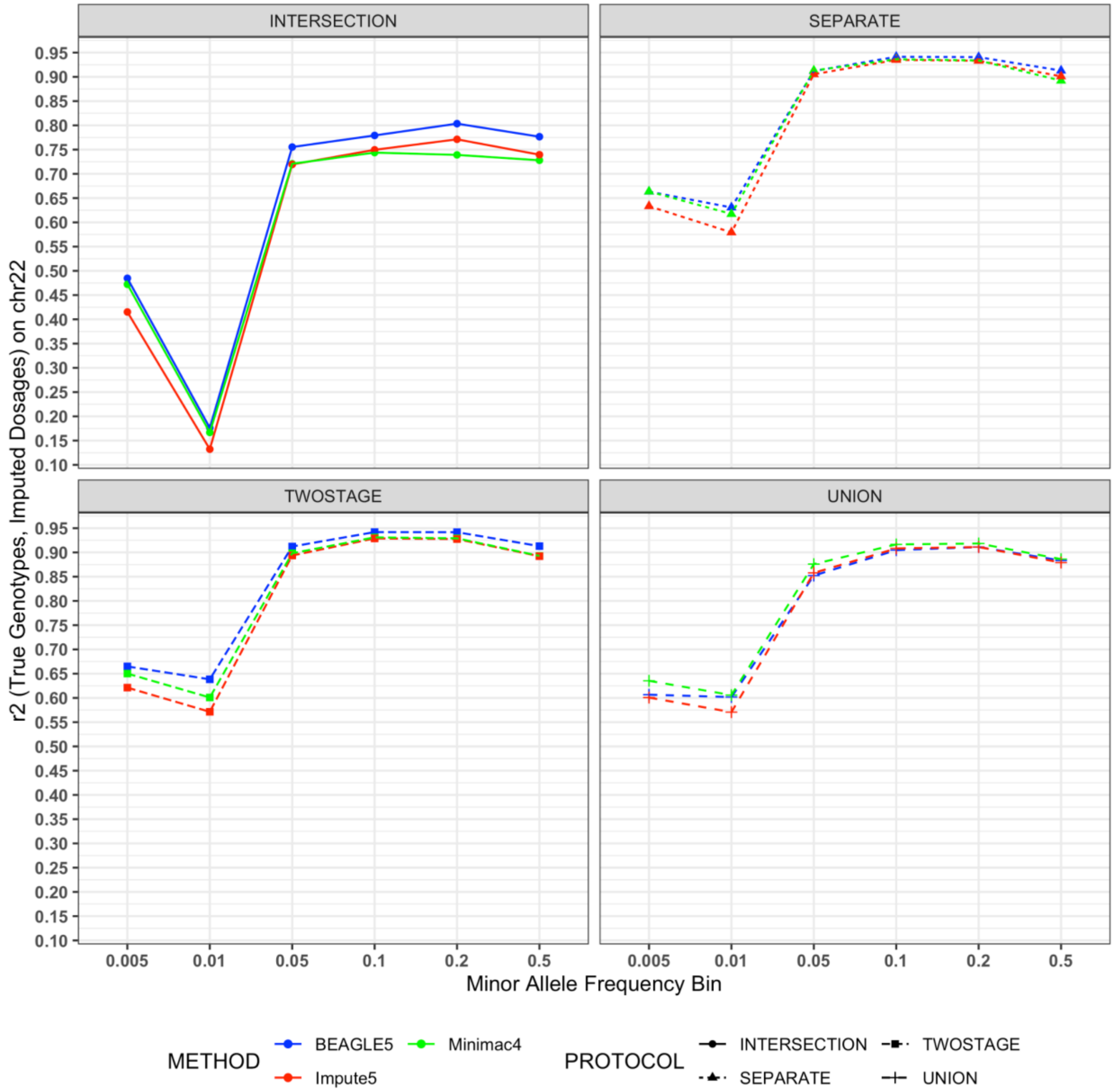

Comparison of imputation accuracies from three different imputation tools across the four different data integration protocols (Blue: Beagle5, Green: Minimac4, Red: Impute5, Solid circle: Intersection protocol, Dashed Square: Twostage protocol, Dashed triangle: Separate protocol, Dashed plus sign: Union protocol). Pre-phasing was done using BEAGLE5.1.

Supplementary Figure 4. Accuracy of imputation varies by parental origin and across data integration protocols.

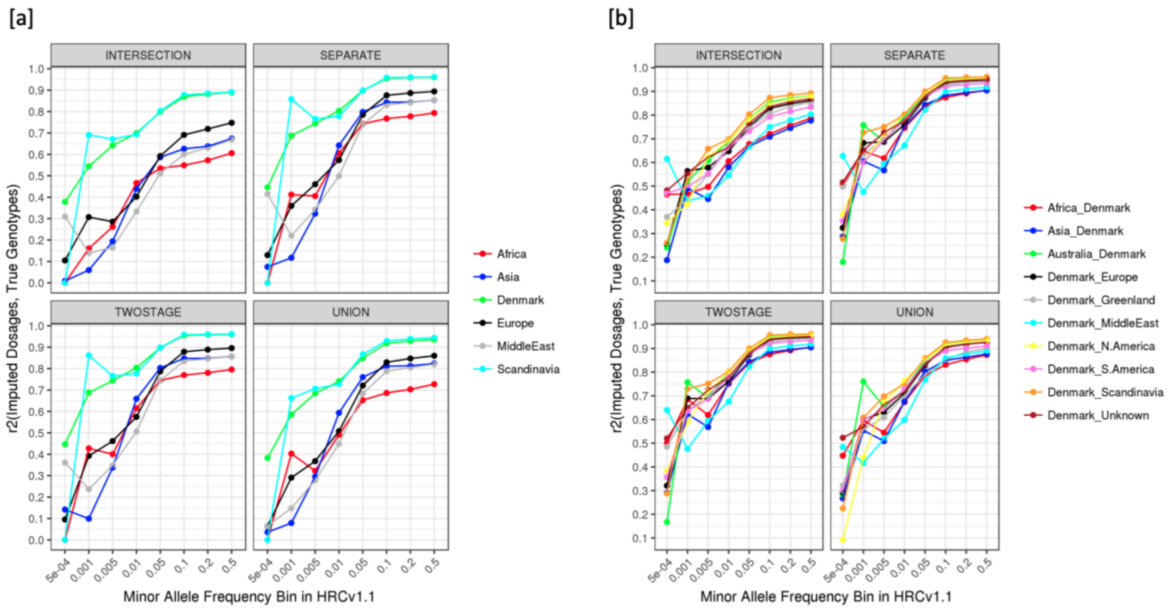

The attenuation in imputation accuracy within samples of non-European origin is further magnified by choice of data integration protocol. [a] Shows the accuracy of imputation within the 10,000 masked SNPs at different minor allele frequency bins within samples grouped by the birthplace of their parents according to the Danish civil registers across all four data integration protocols. (Both parents born in Africa: red, Asia: blue, Denmark: green, European non-Danish or Scandinavian: black, Middle East: gray, Scandinavian non-Danish: cyan) [b] Shows the accuracy of imputation within the 10,000 masked SNPs within samples where at least one parent was born in Denmark. (Non-Danish parent born in Africa: red, Asia: blue, Australia: green, Europe non-Scandinavian or Danish: black, Greenland: gray, Middle East: Cyan, North America: yellow, South America: pink, Scandinavia non-Danish: orange, Unknown: brown).

Supplementary Note 3

To evaluate the impact of the quality of phasing and imputations on power in genome wide association studies, we performed an association study with the simulated continuous phenotype (see Methods) regressed against the true and imputed genetic markers at the 10,000 SNPs masked after QC and prior to phasing (see Methods) within the unrelated individuals of a homogeneous genetic subset of individuals (Supplementary S1.1) in iPSYCH2012, 2015i and the combined cohort. The genomic control coefficient (λ_{gc}) calculated from these summary statistics is presented in Supplementary Table 6 and Figure 5. As the phenotype simulations involved specifying all 10,000 loci as causal SNPs, the λ_{gc} in this scenario should be interpreted as the degree to which the imputed genotypes recover the polygenicity and power in an association test as compared to using the true genotypes. The results suggest that the loss of power in the association study follows the quality of phasing and imputations arising from each data integration protocol with the more accurate separate and twostage protocols suffering less loss in power as compared to the union and intersection protocols.

COHORT	PROTOCOL	LAMBDA_GC
iPSYCH2012	SEPARATE	4.67
iPSYCH2012	UNION	4.59
iPSYCH2012	INTERSECTION	4.28
iPSYCH2012	TWOSTAGE	4.65
iPSYCH2012	TRUTH	4.87
iPSYCH2015i	SEPARATE	3.22
iPSYCH2015i	UNION	3.16
iPSYCH2015i	INTERSECTION	3.04
iPSYCH2015i	TWOSTAGE	3.24
iPSYCH2015i	TRUTH	3.35
iPSYCH2015	SEPARATE	6.75
iPSYCH2015	UNION	6.52
iPSYCH2015	INTERSECTION	6.21
iPSYCH2015	TWOSTAGE	6.76
iPSYCH2015	TRUTH	7.1

Supplementary Table 2. Genomic Control calculated from association test statistics obtained by regressing a simulated continuous phenotype against genotypes within iPSYCH2012, 2015i and the combination of the two, iPSYCH2015 showing the loss of power when using imputed dosages from each data integration protocol as compared to using true genotypes.

Supplementary Figure 5. Genomic control coefficients calculated from GWAS of the simulated continuous trait across the four data integration protocols and true genotypes.

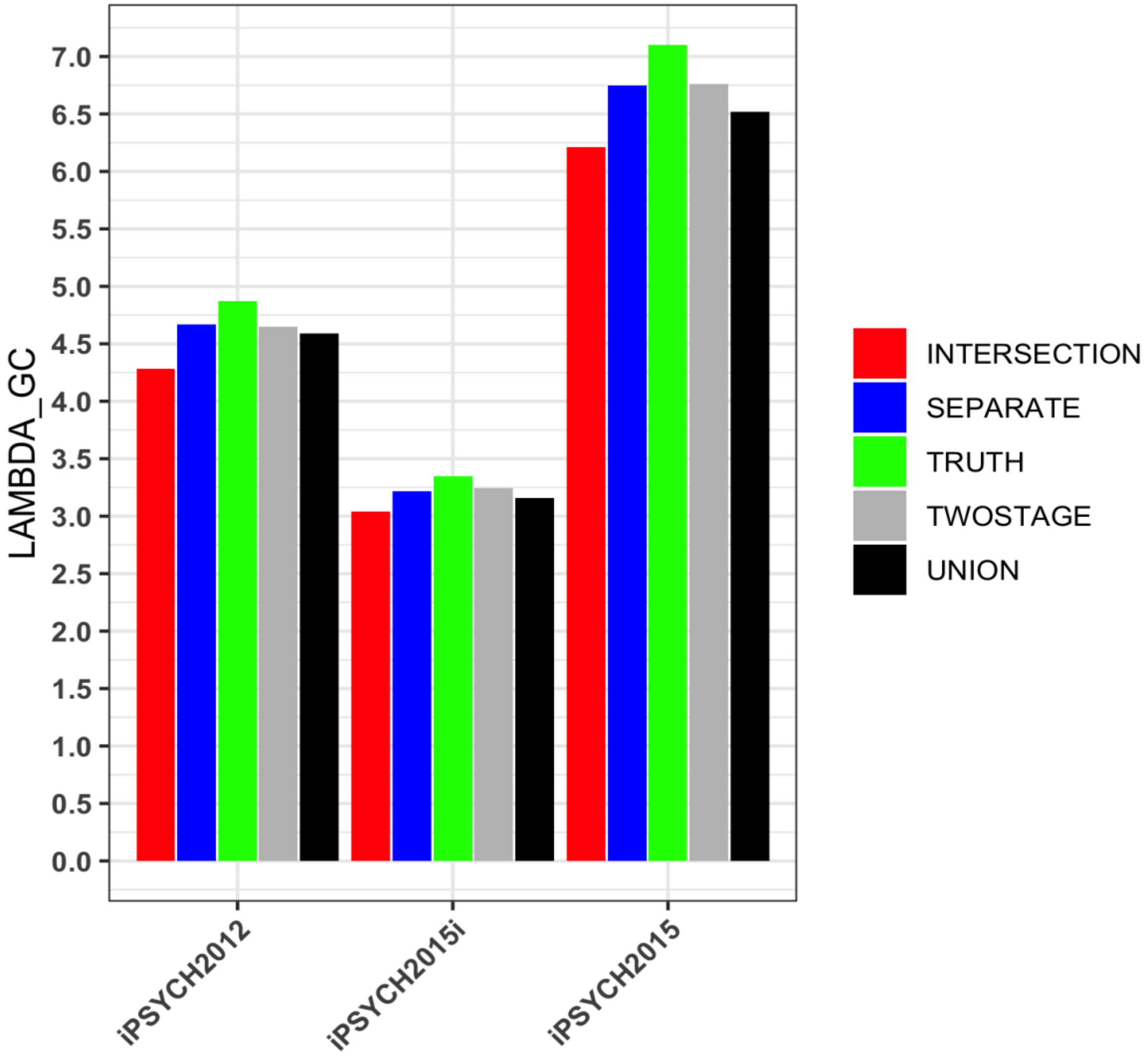

The Genomic control coefficient, as a measure of the loss of power and capability to recover the polygenic signal, calculated from the association statistics obtained by regressing the simulated continuous phenotype against the true genotypes and imputed dosages across all four data integration protocols (intersection: red bars, separate: blue bars, true genotypes: green bars, twostage: gray bars, union: black bars) in iPSYCH2012, 2015i and the combined cohort at the 10,000 masked causal markers used in the simulations.

Supplementary Note 4

Reliability of imputation quality metrics

Imputation software, such as BEAGLE5.1 provides an estimated quality score for imputed dosages (BEAGLE-r^2) at each SNP, which is a predicted correlation between the true and estimated genotypes at a given variant. The r^2 at an imputed locus is an important quantity, as it can be used to estimate the reduction in effective sample size for an association test[3] and as a filtering threshold to ensure only high quality markers are used for association tests and polygenic scoring[4]. We sought to evaluate the robustness of this metric across data integration protocols by comparing it to the empirical imputation accuracy (Empirical-r^2) calculated from the 10,000 masked SNPs (Supplementary Figure 3). The squared Pearson correlation coefficient of BEAGLE-r^2 and EMPIRICAL-$r^2$is highest for intersection protocol ($r^2_{BEAGLE-r2, EMPIRICAL-r2} = 0.98$) protocol and lowest for the union ($r^2_{BEAGLE-r2, EMPIRICAL-r2} = 0.77$) (Supplementary Figure 3). Hence, uncertainties introduced by high genotype missingness in the target dataset, prior to phasing travels through the whole genome imputation pipeline, leading to a potential inclusion of genotype dosages, estimated at less than the recommended thresholds and impacting the accuracy of estimates and replicability of complex trait analyses.

Supplementary Figure 6. Robustness of the imputation quality metric emitted by BEAGLE5 across data integration protocols.

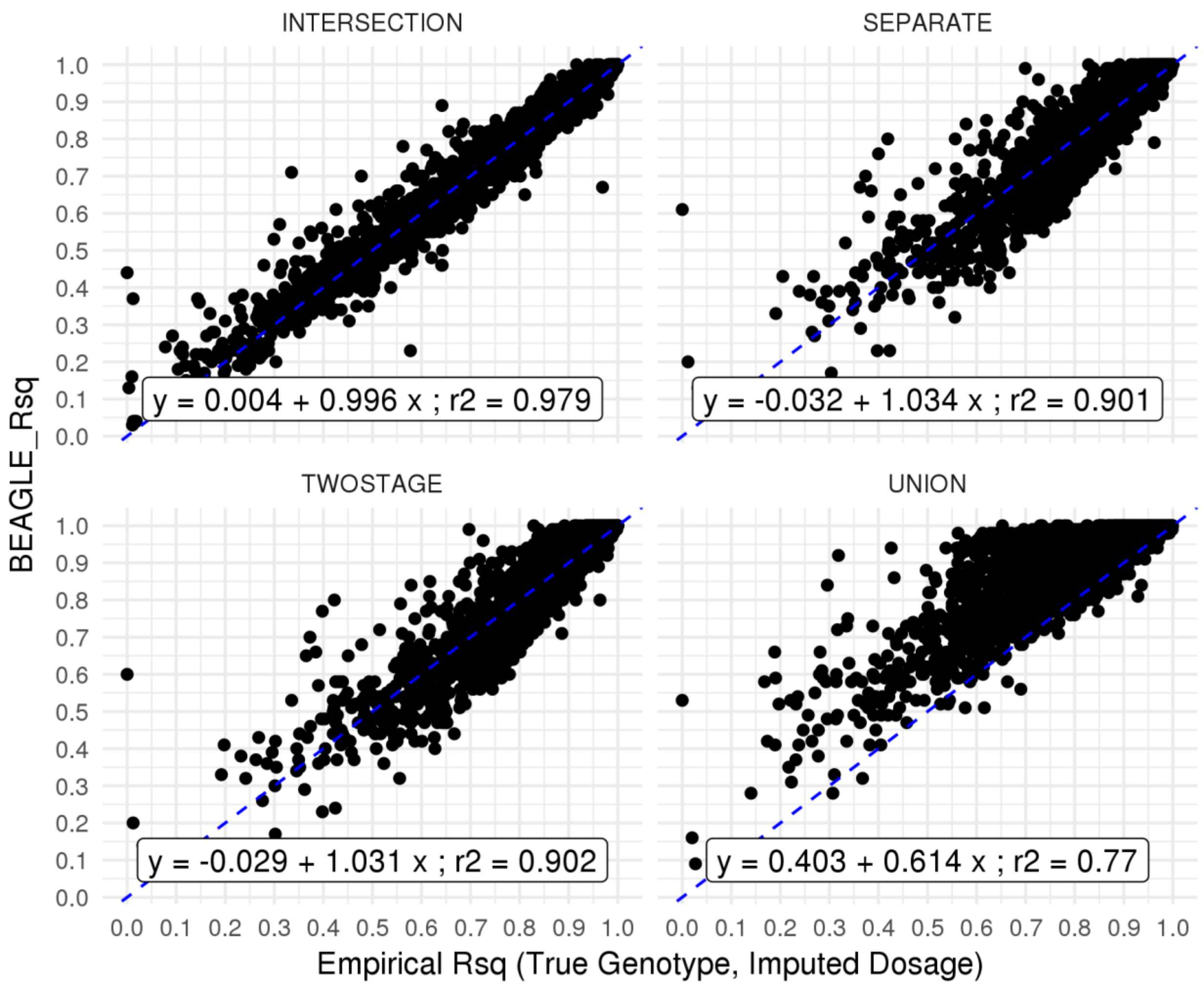

The relationship between empirical imputation accuracy, as measured by the squared Pearson correlation coefficient of true genotypes and imputed dosages at 10,000 masked SNPs, and BEAGLE r^2 within each data integration protocol. The plot shows the BEAGLE r^2 is best calibrated for the imputations from the intersection protocol whereas it overestimates the accuracy, in presence of high genotype missingness, as present in the union protocol. Dashed blue line indicates a slope = 1.

SUPPLEMENTARY REFERENCES

1. Pedersen, C. B. The Danish Civil Registration System. *Scand. J. Public Health* **39**, 22–25 (2011).
2. Chervova, O. *et al.* The Personal Genome Project-UK, an open access resource of human multi-omics data. *Sci Data* **6**, 257 (2019).
3. Pritchard, J. K. & Przeworski, M. Linkage disequilibrium in humans: models and data. *Am. J. Hum. Genet.* **69**, 1–14 (2001).
4. Choi, S. W., Mak, T. S. & O'Reilly, P. F. Tutorial: a guide to performing polygenic risk score analyses. *Nat. Protoc.* **15**, (2020).
5. Rubinacci, S., Delaneau, O. & Marchini, J. Genotype imputation using the Positional Burrows Wheeler Transform. *Cold Spring Harbor Laboratory* 797944 (2020) doi:10.1101/797944.
6. Das, S. *et al.* Next-generation genotype imputation service and methods. *Nat. Genet.* **48**, 1284–1287 (2016).
7. Browning, B. L., Zhou, Y. & Browning, S. R. A One-Penny Imputed Genome from Next-Generation Reference Panels. *Am. J. Hum. Genet.* **103**, 338–348 (2018).
