# Supplementary material for: Accuracy of haplotype estimation and whole genome imputation affects complex trait analyses in complex biobanks
Source: Commun Biol. 2023 Jan 26;6:101. doi: 10.1038/s42003-023-04477-y (PMC9876938; doi:10.1038/s42003-023-04477-y)
Supplement: Supplementary file 3 — Description of Additional Supplementary Data [file 42003_2023_4477_MOESM3_ESM.docx]

**Description of Additional Supplementary Files**

**File name:** Supplementary Data 1

**Description:** The source data behind Figure 2a.

**File name:** Supplementary Data 2

**Description:** The source data behind Supplementary Figure 1.

**File name:** Supplementary Data 3

**Description:** The source data behind Figure 2b.

**File name:** Supplementary Data 4

**Description:** The source data behind Supplementary Figure 2.

**File name:** Supplementary Data 5

**Description:** The source data behind Supplementary Figure 3.

**File name:** Supplementary Data 6

**Description:** The source data behind Figure 3 and Supplementary Figure 4.

**File name:** Supplementary Data 7

**Description:** The source data behind Figure 4a.

**File name:** Supplementary Data 8

**Description:** The source data behind Figure 4b.

**File name:** Supplementary Data 9

**Description:** The source data behind Figure 5.
