# Supplementary material for: Accuracy of haplotype estimation and whole genome imputation affects complex trait analyses in complex biobanks
Source: Commun Biol. 2023 Jan 26;6:101. doi: 10.1038/s42003-023-04477-y (PMC9876938; doi:10.1038/s42003-023-04477-y)
Supplement: Supplementary file 13 — Reporting Summary-New [file 42003_2023_4477_MOESM13_ESM.pdf]

nature portfolio

Corresponding author(s): Vivek Appadurai | vivek.appadurai@regionh.dk

Last updated by author(s): Dec 8, 2022

Reporting Summary

Nature Portfolio wishes to improve the reproducibility of the work that we publish. This form provides structure for consistency and transparency in reporting. For further information on Nature Portfolio policies, see our Editorial Policies and the Editorial Policy Checklist.

Statistics

For all statistical analyses, confirm that the following items are present in the figure legend, table legend, main text, or Methods section.

n/a	Confirmed	
☐	☒	The exact sample size (*n*) for each experimental group/condition, given as a discrete number and unit of measurement
☐	☒	A statement on whether measurements were taken from distinct samples or whether the same sample was measured repeatedly
☐	☒	The statistical test(s) used AND whether they are one- or two-sided<br>*Only common tests should be described solely by name; describe more complex techniques in the Methods section.*
☐	☒	A description of all covariates tested
☐	☒	A description of any assumptions or corrections, such as tests of normality and adjustment for multiple comparisons
☐	☒	A full description of the statistical parameters including central tendency (e.g. means) or other basic estimates (e.g. regression coefficient) AND variation (e.g. standard deviation) or associated estimates of uncertainty (e.g. confidence intervals)
☐	☒	For null hypothesis testing, the test statistic (e.g. *F*, *t*, *r*) with confidence intervals, effect sizes, degrees of freedom and *P* value noted<br>*Give P values as exact values whenever suitable.*
☒	☐	For Bayesian analysis, information on the choice of priors and Markov chain Monte Carlo settings
☒	☐	For hierarchical and complex designs, identification of the appropriate level for tests and full reporting of outcomes
☐	☒	Estimates of effect sizes (e.g. Cohen's *d*, Pearson's *r*), indicating how they were calculated

Our web collection on statistics for biologists contains articles on many of the points above.

Software and code

Policy information about availability of computer code

Data collection	External data sources were downloaded using the UNIX command wget.
Data analysis	Haplotype estimations were carried out using BEAGLE5.1, SHAPEIT4.1.2, EAGLE2.4.1. Missing genotype data imputations were carried out by BEAGLE5.1. Data manipulations and cleaning were performed using PLINK2, GENOTYPE HARMONIZER 1.4.20, bcftools1.9. Kinship QC was performed using KING. Data analysis and plotting were done using R versions 3.3.1, 4.1.0 with dplyr and ggplot packages. Principal components were computed using flashpca2.0. Switch error rates to evaluate haplotype estimation accuracy were calculated using code: https://github.com/SPG-group/switchError Imputation accuracies were evaluated using code: https://github.com/vaqm2/impute_paper

For manuscripts utilizing custom algorithms or software that are central to the research but not yet described in published literature, software must be made available to editors and reviewers. We strongly encourage code deposition in a community repository (e.g. GitHub). See the Nature Portfolio guidelines for submitting code & software for further information.

Data

Policy information about availability of data

All manuscripts must include a data availability statement. This statement should provide the following information, where applicable:

- Accession codes, unique identifiers, or web links for publicly available datasets
- A description of any restrictions on data availability
- For clinical datasets or third party data, please ensure that the statement adheres to our policy

In accordance with the consent structure governing iPSYCH data, the genotype and phenotype information cannot be made available for public access. However, any intermediate level data can be made available upon request and followed by an ethical review. The UK personal genomes project data used in this paper can be downloaded from the European nucleotide archive with the accession code: PRJEB17529. Haplotype reference consortium v1.1 and 1000 Genomes phase3 callsets were used as reference datasets and are available for public download. All accuracy measures mentioned in the results and plots have been included in the manuscript and supplementary tables.

Human research participants

Policy information about studies involving human research participants and Sex and Gender in Research.

Reporting on sex and gender	As the analysis presented in this research was primarily concerned with bioinformatics, computational accuracy and data integration protocols, no sex and gender specific analyses was conducted. In general, the gender information for iPSYCH is gleaned from the Danish Birth registers.
Population characteristics	Samples collected as part of iPSYCH (N = 134690) were singleton births that occurred between 1981 and 2007 with a known mother, alive and residing in Denmark at the end of their first year of birth. Cases were ascertained for individuals born with one of six severe mental disorders, cohort members were a random representative population of Denmark from the same time period.
Recruitment	Samples were recruited on a consent by non opt-out basis from a national research program. Corresponding DNA was extracted from dried blood spots taken from The Danish National Neonatal Screening Biobank.
Ethics oversight	iPSYCH was approved by The Central Denmark Region Committee on Health Research ethics. Use of iPSYCH data is governed by The Danish Scientific Ethics Committee, The Danish Health Authority, Danish data protection agency and the Danish Neonatal Screening Biobank Committee. Personal Genomes Project UK has been approved by the University College London scientific ethics committee.

Note that full information on the approval of the study protocol must also be provided in the manuscript.

Field-specific reporting

Please select the one below that is the best fit for your research. If you are not sure, read the appropriate sections before making your selection.

☒ Life sciences ☐ Behavioural & social sciences ☐ Ecological, evolutionary & environmental sciences

For a reference copy of the document with all sections, see nature.com/documents/nr-reporting-summary-flat.pdf

Life sciences study design

All studies must disclose on these points even when the disclosure is negative.

Sample size	The sample size (N = 134690) includes all samples genotyped as part of iPSYCH, as of 2018 and passing initial technical QC.
Data exclusions	Samples of a non-European origin were excluded where appropriate for genetic analyses such as polygenic scores using a pruning process guided by principal component analysis. To evaluate biases in association tests, we further restricted analyses to the randomly ascertained cohort of iPSYCH to avoid flagging possible allele frequency differences between cases and controls at trait associated loci as false positives.
Replication	The research is mostly empirical although the trends generally translate between the two cohorts of iPSYCH and the Personal Genomes Projects UK, wherever possible.
Randomization	As we are only concerned with computational accuracy of bioinformatics tools and processes, no sample randomization was performed. For SNP masking, to evaluate imputation accuracy, we randomly assigned effect sizes using GCTA following a standard normal distribution over SNPs selected using 'sample' function in R.
Blinding	As research is mostly concerned with empirical comparisons of bioinformatics and computational approaches, no blinding was necessary.

Reporting for specific materials, systems and methods

We require information from authors about some types of materials, experimental systems and methods used in many studies. Here, indicate whether each material, system or method listed is relevant to your study. If you are not sure if a list item applies to your research, read the appropriate section before selecting a response.

Materials & experimental systems

n/a	Involved in the study
☒	☐ Antibodies
☒	☐ Eukaryotic cell lines
☒	☐ Palaeontology and archaeology
☒	☐ Animals and other organisms
☒	☐ Clinical data
☒	☐ Dual use research of concern

Methods

n/a	Involved in the study
☒	☐ ChIP-seq
☒	☐ Flow cytometry
☒	☐ MRI-based neuroimaging
